# CD4-Positive T Cells and M2 Macrophages Dominate the Peritoneal Infiltrate of Patients with Encapsulating Peritoneal Sclerosis

**DOI:** 10.1371/journal.pone.0120174

**Published:** 2015-04-24

**Authors:** Sayed M. Habib, Alferso C. Abrahams, Mario R. Korte, Robert Zietse, Lisette L. de Vogel, Walther H. Boer, Amélie Dendooven, Marian C. Clahsen-van Groningen, Michiel G. H. Betjes

**Affiliations:** 1 Department of Internal Medicine, Division of Nephrology and Transplantation, Erasmus Medical Center, Rotterdam, The Netherlands; 2 Department of Nephrology and Hypertension, University Medical Center Utrecht, Utrecht, The Netherlands; 3 Department of Internal Medicine, Division of Nephrology, Albert Schweitzer Hospital, Dordrecht, The Netherlands; 4 Department of Pathology, Erasmus Medical Center, Rotterdam, The Netherlands; 5 Department of Pathology, University Medical Center Utrecht, Utrecht The Netherlands; Kawasaki Medical School, JAPAN

## Abstract

**Background:**

Encapsulating peritoneal sclerosis (EPS) is a severe complication of peritoneal dialysis (PD). Previously, it has been shown that infiltrating CD4-positive T cells and M2 macrophages are associated with several fibrotic conditions. Therefore, the characteristics of the peritoneal cell infiltrate in EPS may be of interest to understand EPS pathogenesis. In this study, we aim to elucidate the composition of the peritoneal cell infiltrate in EPS patients and relate the findings to clinical outcome.

**Study Design, Setting, and Participants:**

We studied peritoneal membrane biopsies of 23 EPS patients and compared them to biopsies of 15 PD patients without EPS. The cellular infiltrate was characterized by immunohistochemistry to detect T cells(CD3-positive), CD4-positive (CD4+) and CD8-positive T cell subsets, B cells(CD20-positive), granulocytes(CD15-positive), macrophages(CD68-positive), M1(CD80-positive), and M2(CD163-positive) macrophages. Tissues were analysed using digital image analysis. Kaplan-Meier survival analysis was performed to investigate the survival in the different staining groups.

**Results:**

The cellular infiltrate in EPS biopsies was dominated by mononuclear cells. For both CD3 and CD68, the median percentage of area stained was higher in biopsies of EPS as opposed to non-EPS patients (p<0.001). EPS biopsies showed a higher percentage of area stained for CD4 (1.29%(0.61-3.20)) compared to CD8 (0.71%(0.46-1.01), p = 0.04), while in the non-EPS group these cells were almost equally represented (respectively 0.28%(0.05-0.83) *versus* 0.22%(0.17-0.43), p = 0.97). The percentage of area stained for both CD80 and CD163 was higher in EPS than in non-EPS biopsies (p<0.001), with CD163+ cells being the most abundant phenotype. Virtually no CD20-positive and CD15-positive cells were present in biopsies of a subgroup of EPS patients. No relation was found between the composition of the mononuclear cell infiltrate and clinical outcome.

**Conclusions:**

A characteristic mononuclear cell infiltrate consisting of CD4+ and CD163+ cells dominates the peritoneum of EPS patients. These findings suggest a role for both CD4+ T cells and M2 macrophages in the pathogenesis of EPS.

## Introduction

Encapsulating peritoneal sclerosis (EPS) is a rare and severe complication of peritoneal dialysis (PD) [1,2]. EPS is characterized by fibrotic and sclerotic thickening of the peritoneal membrane (PM), covering and constricting the intestines.[3–5] Although an interplay between chemical irritation of the PM, chronic inflammation, and excessive extracellular matrix deposition may be important, the exact pathogenesis of EPS remains poorly understood.[6,7] Early studies have provided evidence to the hypothesis that inflammation precedes the development of EPS[8,9] offering a window of opportunity to use immunosuppressive therapy in the management of these patients. Declining cancer antigen 125 effluent concentrations (reflecting loss of mesothelial cell mass) and increasing effluent interleukin-6 concentrations have also been linked to EPS development. [10] Recently, we have shown increasing C-reactive protein levels in the months preceding the diagnosis of EPS, which then rise with a maximum at time of clinical diagnosis. [9] These findings indicate the presence of an inflammatory response at the level of the PM, which in turn may drive the progression to excessive fibrotic tissue formation characteristic for EPS.

Although it has been suggested that mononuclear immune cell infiltration is an important hallmark of EPS pathology[11,12], the peritoneal inflammatory cell population in EPS has been poorly defined. This is relevant, as several studies have shown the importance of T cells in regulating inflammatory host responses in fibrotic tissue disorders such as pulmonary fibrosis[13], systemic sclerosis[14], and hepatic fibrosis[15]. In an experimental model, Chung *et al*. [16] have reported an increase of activated T cells homing to the peritoneal cavity shortly after peritoneal injury and proposed a central role for CD4 positive (CD4+) T cells in peritoneal adhesion formation. Furthermore, macrophages are believed to be key cells linking inflammation and fibrosis. Macrophages can be divided into the pro-inflammatory M1 or pro-fibrotic M2 phenotype. [17] Particularly, M2 macrophages have gained interest in recent years as main regulators of fibrosis through secretion of pro-fibrotic mediators and subsequent activation of collagen producing cells.[18]

The main objective of the present study was to characterize the inflammatory cells, in particular T cell and macrophage subsets, in the PM of patients with established EPS in comparison to PD patients without EPS. In addition, we investigated whether the composition of the cell infiltrate was related to clinical outcome of patients.

## Methods

### Study population

We studied PM biopsies of 23 EPS patients and compared them with biopsies of a reference group that consisted of 15 patients who were on PD, but had no EPS (non-EPS). The PM biopsies of EPS patients were obtained during abdominal surgery for diagnostic purposes. All biopsies were collected from the participating centers and stored in the bio-bank of the Dutch EPS registry.[19] PM biopsies in the non-EPS group were performed in PD patients prior to kidney transplantation procedure at the University Medical Center Utrecht, Utrecht, The Netherlands. Patients who had peritoneal infections at time of PM biopsy were excluded from further study.

The diagnosis of EPS was made according to the criteria developed by the Ad Hoc Committee of the International Society for Peritoneal Dialysis. [20] EPS was defined as a clinical syndrome with symptoms of intestinal obstruction, with or without elevated inflammation parameters, and the presence of compatible radiological or macroscopic findings such as peritoneal thickening, calcifications, and encapsulation. Clinical information of patients was gathered by reviewing the medical records. The study was performed with the approval of the Medical Ethics Committee (METC) of the Erasmus Medical Center, Rotterdam, The Netherlands. Approval for performing PM biopsies in PD patients without EPS was obtained from the METC of the University Medical Center Utrecht, Utrecht, The Netherlands. All PD patients gave written informed consent for participation in this study.

### Immunohistochemistry and analysis

Four micrometer sections were cut from the formalin fixed paraffin-embedded tissue. Initially, we performed a haematoxylin-eosin staining on all tissue sections. To determine the type of the mononuclear immune cells, immunohistochemistry was performed on serial sections of the PM biopsies by routine diagnostics on the Benchmark Ultra stainer (Ventana), using antibodies validated for diagnostics and buffers provided by Ventana. Antibodies against cell surface antigens of interest were used to identify mononuclear cells including T cells, macrophages, and their subtypes.

Antibodies against CD3 (1:150 dilution, DAKO, Glostrup, Denmark), CD4 (ready to use, Ventana, Tucson, USA), and CD8 (1:50, DAKO, Glostrup, Denmark) were used to detect all pan-T lymphocytes and T-cell subsets. Antibody against CD68 (1:1600, KP-1, DAKO, Glostrup, Denmark) was used to detect pan-macrophages. In order to determine the subtype of macrophages, we stained biopsies with antibodies against M1 or M2 macrophages. The CD80 antigen has been used as a marker for the detection of M1 macrophages (1:400, R&D, Minneapolis, USA). [17,21] The CD163 antigen, which is known as a haemoglobin scavenger receptor and strongly expressed on M2 macrophages has been used as a marker for the detection of M2 macrophages (1:200, Leica, Wetzlar, Germany). [17,22]

The biopsies were stained simultaneously to reduce inter-staining variation. Incubation with antibodies was done for 32 minutes and anti-rabbit or anti-mouse amplifiers were used. As positive controls, tonsil sections (for CD3, CD4, CD8, CD68, and CD80) and tonsil and lung sections (for CD163) were used. In a subpopulation of seven EPS patients, granulocytes and B cell infiltrates were investigated by staining tissue sections with antibodies against CD15 and CD20 respectively. However, our preliminary findings showed a minute numbers of these cells present, if even any at all, in EPS biopsies. Our focus was therefore only on the presence of T-cells and macrophages.

After staining, the overall histomorphological quality of the tissues was evaluated by two observers who were unaware of the group to which the peritoneal biopsies belonged. All slides were scanned and digitalized on a NDP Nanozoomer virtual microscopy system (Hamamutsu Photonics, Japan). Five random microscopic fields of the submesothelial compact zone in each slide were selected for immunohistochemical analysis using the 40 times power objective (original magnification x400). The submesothelial compact zone was defined as the zone between the basal mesothelial layer and the upper margin of the peritoneal adipose tissue.

Immunohistochemical quantification was performed in a blinded manner using automatic digital image analysis of the slides (KS-400 version 3, 1997, Carl Zeiss Vision GmbH) as previously described and validated.[23] To analyze the immunohistochemical staining for each marker, the amount of staining area was measured across the fields and expressed as a percentage of area stained.

### Statistical analysis

The SPSS software version 20.0 was used for all statistical analysis. Medians (25^th^-75^th^ percentile) are presented of continuous variables and compared using the non-parametric Mann-Whitney test. Categorical variables are presented as numbers and/or percentages, and proportions were tested using the χ²-test. The Spearman’s rank correlation test was used to assess correlation coefficients. Univariate Kaplan-Meier survival analysis was used to investigate the survival in the different staining groups. All probabilities were two tailed. P-values of less than or equal to 0.05 were considered statistically significant.

## Results

### Patient characteristics

The clinical characteristics of the study population are summarized in Table 1. The median age in the EPS group was 42.6 (31.8–61.5) years (*versus* 56.0 (46.0–62.0) years in the non-EPS group, P = 0.11), and the majority of EPS patients were male. EPS patients had a longer cumulative duration of PD as compared to non-EPS patients (58.2 (34.1–77.2) *versus* 30.0 (15.0–41.0) months, P<0.001). The two groups did not significantly differ regarding the incidence of peritonitis or time between last peritonitis and biopsy. Eight EPS patients had a functioning kidney transplant, five EPS patients were on PD, and 10 patients were on hemodialysis (HD) at time of diagnosis.

**Table 1:** Patient characteristics.

	EPS (23)	Non-EPS (15)	P-value
**Age (years)**	42.6 (31.8–61.5)	56.0 (46.0–62.0)	0.11
**Gender (male/female)**	15/8	6/9	0.13
**Cause of renal disease**			-
Glomerulonephritis	8 (34.8)	4 (26.7)	
Interstitial nephritis	4 (17.4)	0 (0.0)	
Renal vascular disease	5 (21.7)	2 (13.3)	
Diabetic nephropathy	2 (8.7)	0 (0.0)	
Cystic kidney disease	0 (0.0)	4 (26.7)	
Congenital/Hereditary kidney disease	2 (8.7)	0 (0.0)	
Other	2 (8.7)	2 (13.3)	
Unknown	0 (0.0)	3 (20.0)	
**Cumulative peritoneal dialysis (months)**	58.2 (34.1–77.2)	30.0 (15.0–41.0)	< 0.001
**History of peritonitis (yes)**	17 (73.9)	8 (53.3)	0.13
**Peritonitis episodes per patient year**	0.42 (0.1–1.4)	0.21 (0.0–1.4)	0.32
**Time since last peritonitis until biopsy (months)**	6.8 (4.6–27.4)	11.3 (7.6–22.7)	0.51

Values are median (25^th^-75^th^ percentile) or n (%). Medians were compared using the non-parametric Mann-Whitney test. Proportions were compared using the χ² - test. P value ≤0.05 was considered significant

### Immunohistochemical analysis

**Fig 1:**
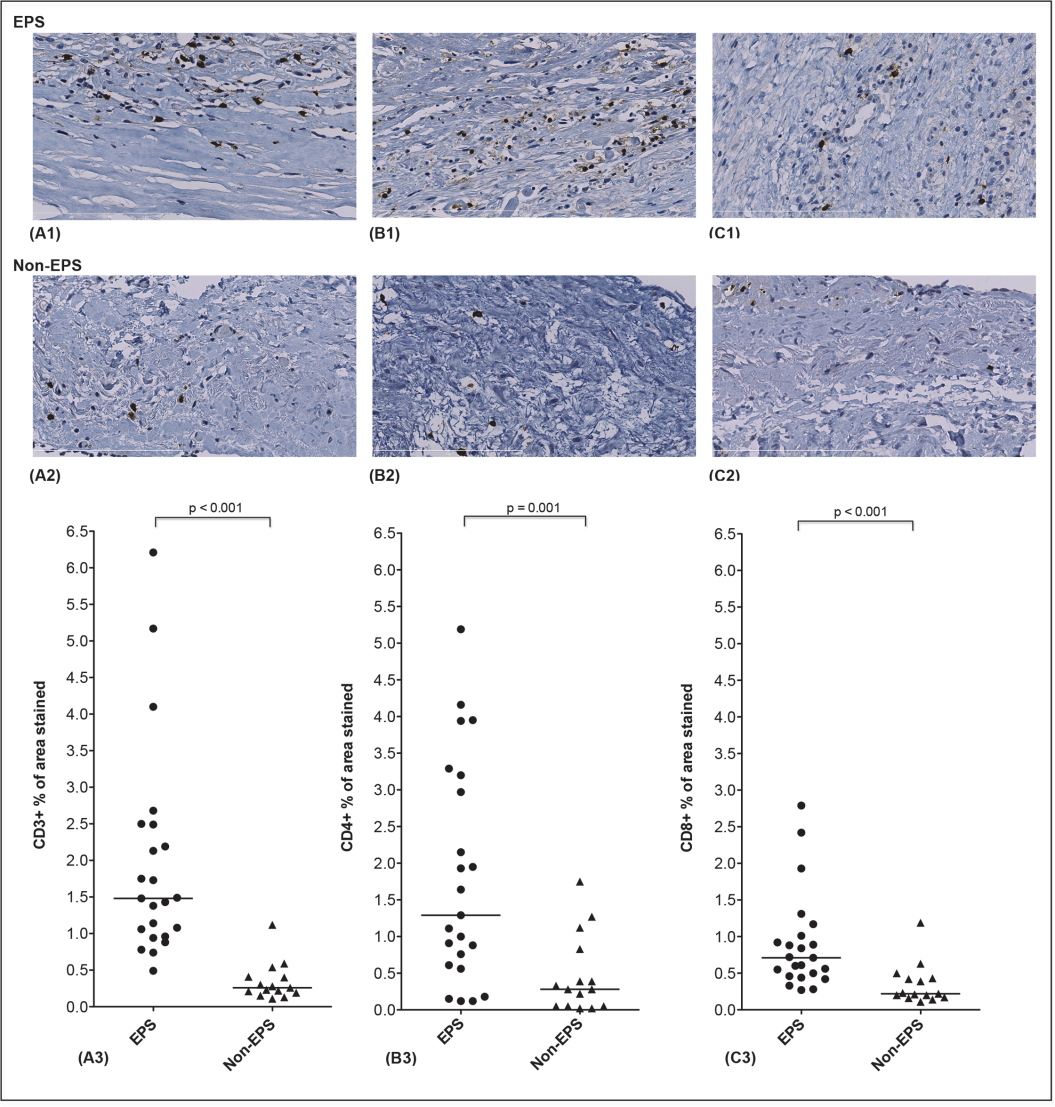
Immunohistological features of the infiltrating T lymphocyte population. Representative peritoneal findings are shown for both groups: (A1-2) CD3+ cell fields (B1-2) CD4+ cell fields (C1-2) CD8+ cell fields. Magnification x400. A quantitative comparison of the percentage of area stained for all markers in the peritoneal membrane of EPS and non-EPS patients is shown in A3, B3, and C3. Medians (25^th^-75^th^ percentile) are presented and compared using the non-parametric Mann-Whitney test. Scale bar shows 200um length.

**Fig 2:**
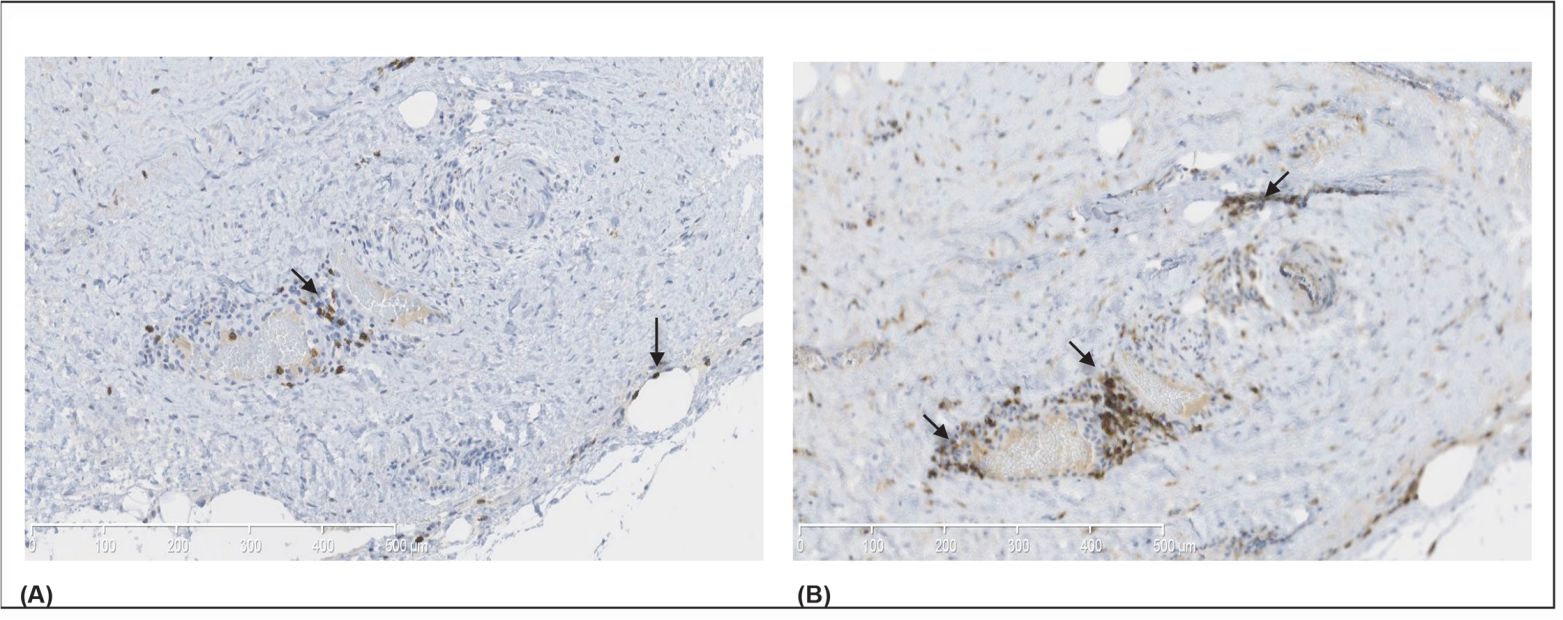
Staining of peritoneum of a patient with antibodies against CD4 and CD8. In 2A a few CD8+ (brown) cells are present in a mononuclear cell infiltrate while in 2B a substantial number of CD4+ cells are identified in the same infiltrate and scattered throughout the thickened and sclerosed submesothelial tissue. Magnification x150. Scale bar shows 500um length.

Immunohistochemical staining was adequate in all biopsies and quantification could be performed for all markers. The cellular infiltration in EPS biopsies was predominated by a mononuclear cell infiltrate that was mainly found scattered and at times patchy throughout the fibrotic submesothelial area. Fig 1 shows the results of immunohistochemistry in both groups for the T cell markers. In patients with EPS, there was an increased percentage of area stained for CD3, as compared to the non-EPS group (Fig 1A3). The percentage of area stained for CD4 (Fig 1B3) and CD8 (Fig 1C3) were also increased in biopsy specimens of EPS patients, as compared to the non-EPS group. Additionally, the difference in percentage of area stained between CD4 and CD8 was analyzed, and an increased percentage of area stained for CD4 in comparison to CD8 (Fig 2) in EPS biopsies was observed. Interestingly, while the difference in CD4 and CD8 percentage of area stained was significant in biopsies of EPS patients (1.29% (0.61–3.20) *versus* 0.71% (0.46–1.01), p = 0.04) this was not the seen in biopsies of the non-EPS group where these cell types were equally represented (0.28% (0.05–0.83) *versus* 0.22% (0.17–0.43), p = 0.97).

**Fig 3:**
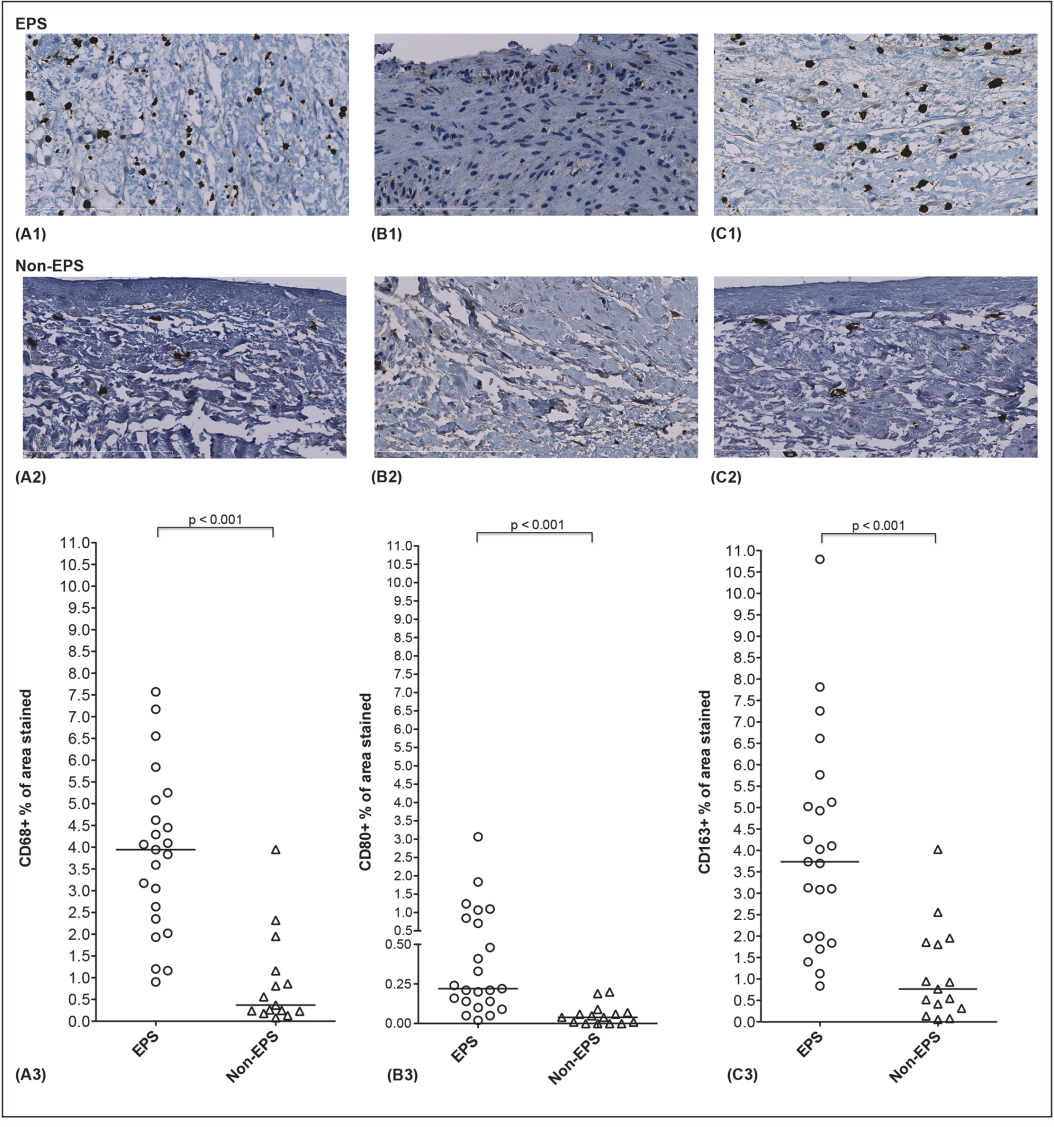
Immunohistological features of infiltrating macrophages. Representative peritoneal findings are shown for both groups: (A1-2) CD68+ cell fields (B1-2) CD80+ cell fields, (C1-2) CD163+ cell fields. Magnification x400. A quantitative comparison of the percentage of area stained for all macrophage markers in the peritoneal membrane of EPS patients and PD controls is shown in A3, B3, and C3. Medians (25^th^-75^th^ percentile) are presented and compared using the non-parametric Mann-Whitney test. Scale bar shows 200um length.

Fig 3 shows the distribution of CD68 staining in both EPS and non-EPS group. CD68+ cells were diffusely distributed along the submesothelial area. We observed a significantly higher percentage of area stained for CD68 in EPS biopsies when compared to the non-EPS group (Fig 3A3). With respect to M1 macrophages, CD80+ staining was sparsely present in EPS biopsies and rarely detected in tissues of the non-EPS group (0.22% (0.14–0.85) *versus* 0.04% (0.00–0.07), P<0.001) (Fig 3B3). In contrast, M2 macrophages, as assessed by CD163+ staining, were the most abundant macrophage phenotype and almost 5 times higher in extent in EPS tissues as compared to the non-EPS group (3.74% (0.20–5.13) *versus* 0.77% (0.32–1.86), P<0.001) (Fig 3C3). In the eight post-transplantation EPS patients who were on steroids, no significant differences in the percentage of area stained for pan-T cells (CD3+ staining: 1.61% (1.20–2.04) *versus* 1.43% (0.88–2.68), p = 0.88) or pan-macrophages (CD68+ staining: 3.89% (2.42–4.48) *versus* 4.09% (2.02–5.84), p = 0.59) were noted as compared to the fifteen patients who were not receiving steroids.

### Peritoneal cellular infiltrate and clinical outcome

**Fig 4:**
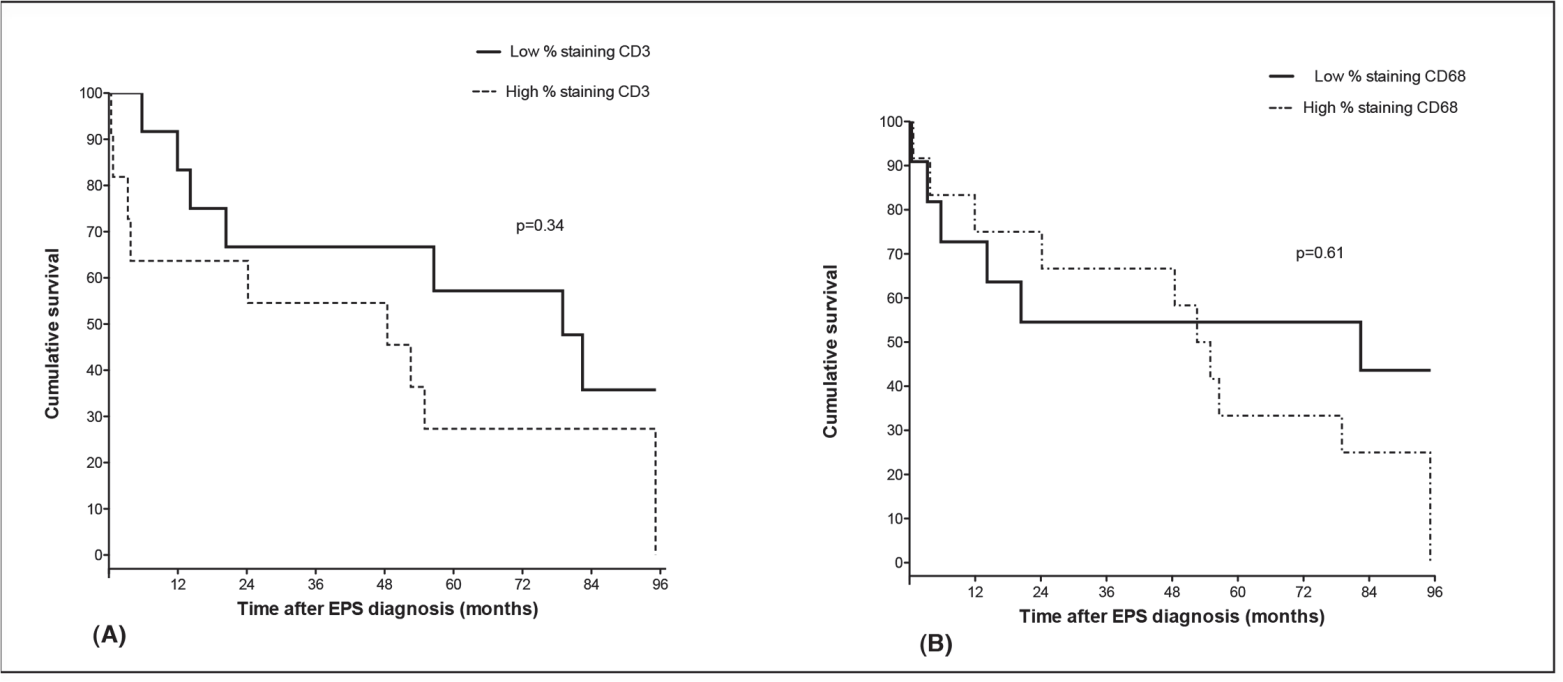
Kaplan-Meier curves showing overall survival comparison between high (dotted line) and low (solid line) staining groups for CD3 (4a) and CD68 (4b). P values are based on the log-rank test.

During follow up (median 52.6 (11.9–85.0) months), 17 (73.9%) out of 23 EPS patients died, with a median time of death of 24.2 (4.76–55.77) months after diagnosis. In order to evaluate a possible influence of T cells and macrophages on the clinical outcome of EPS patients, we divided patients into two staining groups. A group with a percentage of area stained below the corresponding median (low staining) and a group with a percentage of area stained equal or above to the corresponding median (high staining) for CD3 or CD68 staining. As it is shown in Fig 4A, we did not find a significant difference in survival between the low and high staining group for CD3. Similarly, no significant difference in survival was observed in the two groups with regard to the presence of CD68 (Fig 4B). Separate analysis after stratification by the diagnostic subgroup to which the patients belonged to (post-transplantation or classical (being on HD or PD) EPS) did not change these results (data not shown).

**Fig 5:**
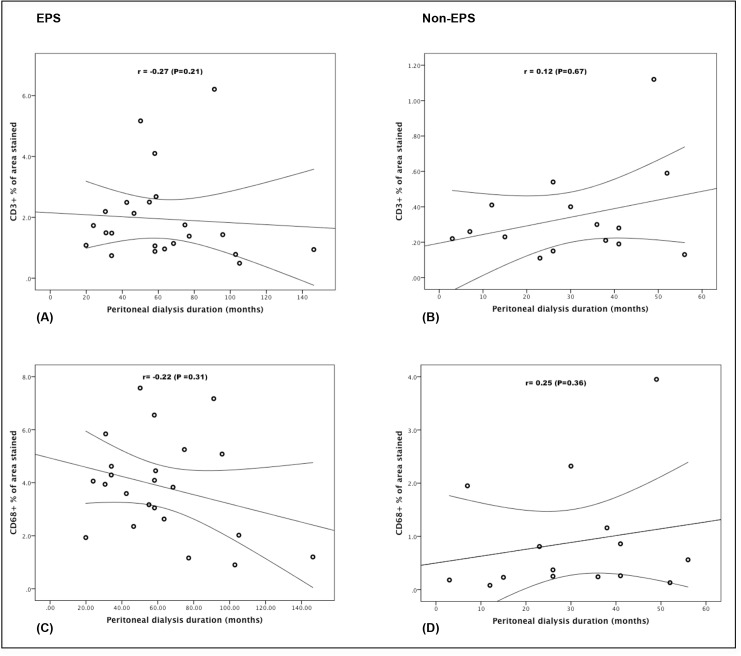
Correlation between PD duration and percentage of area stained for CD3 and CD68 in the EPS (5a and 5c) and the non-EPS group (5b and 5d). In all graphs, the regression line and the 95% confidence interval are plotted.

For further analysis, we also assessed the correlation between the percentage of area stained and PD duration for each one of the markers in both groups, and calculated Spearman’s rank correlation coefficients. Subsequently, a negative correlation between PD duration and percentage of area stained for CD3 (r = -0.27, P = 0.21) and CD68 (r = -0.22, P = 0.31) over time was observed in the EPS group (Fig 5A and 5C), though not statistically significant. In addition, no significant correlations between PD duration and percentage of area stained for CD3 (r = 0.12, P = 0.67) or CD68(r = 0.25, P = 0.36) was found in the non-EPS group (Fig 5B and 5D).

## Discussion

In this study, we found a more intense mononuclear cell infiltration, predominantly consisting of CD4+ cells and CD163+ cells, in the PM of patients with EPS as compared to the presence of these cells in the PM of PD patients without EPS. These findings provide evidence for a local peritoneal inflammatory response in EPS and support the concept of immune cell-mediated excessive fibrosis in the pathogenesis of this condition.

The results of our study are in agreement with previous studies reporting on the presence of chronic inflammation in the PM of patients with EPS. In this context, a study by Honda *et al*.[11] revealed an increased frequency of mononuclear cell infiltration in the EPS group as compared to a non EPS group. The presence of a mononuclear cell infiltrate has also been proposed as one of the histomorphologic criteria for EPS. [11,12] Furthermore, in a mouse model of EPS, histological evaluation of sections from the abdominal cocoon revealed fibrotic thickening of the intestinal serosa with an infiltrate of mononuclear cells and only a few polymorph nuclear leukocytes. [24] However, although these studies underscore the inflammatory nature of EPS, they do not report on the presence of specific subsets of T cells and macrophages, which are known to be involved in profibrotic responses.

In the present study we found an increased extent of staining for both CD4+ and CD8+ cells in EPS tissues in comparison to the non-EPS group tissues. However, the increase in CD4+ cells in EPS tissues was relatively more pronounced as compared to CD8+ cells. Normally, the CD4/CD8 ratio of T cells within the peritoneal effluent of stable PD patients is reported to be decreased (below 1.5) [25], which is not in accordance with the distribution of these cell subsets in the PM of our EPS patients. Therefore, our observations contribute to the hypothesis that recruitment of CD4+ T cells in the PM is of possible importance in the pathogenesis of EPS. CD4+ T cells have a pivotal effect on collagen synthesis by their production of pro-fibrotic cytokines. [26] The production of T-helper-2 (Th-2) cytokines, such as IL-4 and IL-13, may enhance fibrosis formation through activation of M2 macrophages or stimulation of fibroblast proliferation. [13,26] In a mouse model, Zampell *et al*. [27] have reported on the importance of CD4+ cells in the regulation of fibrosis. In this study, CD4+ cell depleted animals showed a decrease in dermal fibrosis as reflected by a decline in type I collagen deposition and dermal scar index. In contrast, non-significant decreases in scar index and no decreases in type I collagen deposition were noted in CD8+ cell depleted mice as compared to controls.

In our study, we have demonstrated an increased presence of macrophages in the PM biopsies of EPS patients. Macrophages play a crucial role in chronic inflammation-induced fibrosis by their production of pro-fibrotic cytokines and ability to regulate extracellular matrix turnover via controlling the balance of several matrix metalloproteases.[28] With their capacity to act pro-fibrotic, M2 macrophages are thought to promote fibrosis through production of transforming growth factor-beta (TGF-β) and stimulation of fibroblasts.[18,28] Consistent with findings in biopsies from patients with other fibrotic diseases, such as pulmonary fibrosis [29], we found a predominance of M2 macrophages in the peritoneal tissue of EPS patients, highlighting their importance. Bellón *et al*.[30] have previously reported on the phenotype of peritoneal effluent macrophages and demonstrated the capacity of CD163+CD14+ cells to stimulate proliferation of human fibroblasts. The capacity of peritoneal macrophages to stimulate fibroblast proliferation correlated strongly with chemokine ligand 18 (CCL18) mRNA levels. Additionally, they showed that an increased concentration of CCL18, a pro-fibrotic chemokine produced by M2 macrophages, was found in the peritoneal effluent of patients who later developed EPS. In support of this finding, a prospective observational study by Ahmad *et al*.[31] has reported higher baseline dialysate and serum levels of CCL18 in patients who developed EPS as compared to a stable PD group.

In the present study, both T cell and macrophage infiltration were not significantly associated with clinical outcome of EPS patients. In addition, we did not observe a difference in immunohistochemical staining between EPS patients who received steroids as part of transplantation immunosuppressive protocol as compared to those patients who were on PD or HD at time of biopsy. With regard to this, and as part of one of the limitations in this study, the low number of patients in the subgroups could have been of influence in demonstrating significant differences. Moreover, the scope of our study was not the fibrotic changes in EPS *per se* but the characterization of the peritoneal cell infiltrate. Therefore, our speculations regarding the association between inflammatory cells and promotion of fibrosis in EPS needs further study.

Another limitation of our study is the difference in PD duration between the two groups, which may have influenced the amount of cell infiltration in the biopsies. Hence, we have focused on the influence of PD duration on the percentage of area stained for CD3 and CD68 in each of the groups and did not observe significant correlations between these two variables. Importantly, other studies have reported a decrease in both lymphocytes in the peripheral blood of PD patients[32,33], and macrophages in the PD effluent of PD patients[25] during the course of PD treatment. Due to the descriptive nature of our study, the exact mechanisms responsible for the increased cellular response that was observed in EPS patients could not be studied and remain uncertain. Yáñez-Mó *et al*. [34] have reported on alterations of the PM and transdifferentiation of peritoneal mesothelial cells into myofibroblasts in PD patients. Considering this, we can speculate that these alterations of the PM may be a consequence to inflammation ending in severe peritoneal inflammation and fibrosis. Most EPS patients have had a long history of PD. The bio-incompatible elements in PD fluids to which the PM mesothelial cells are exposed may increase oxidative stress and induce the production and release of pro-inflammatory mediators that can enhance a CD4+ T helper-2 cell response. CD4+ T helper-2 cell subsets along with the pro-fibrotic cytokine microenvironment in the peritoneal cavity may in turn promote the infiltration of macrophages or polarization of resident macrophages that would be activated towards an M2 type. Finally, these M2 macrophages may further contribute to the fibrotic process development through the secretion of TGF-β or other fibrogenic factors ending in fibroblast proliferation and excessive collagen synthesis. However, additional experimental studies are required to further examine this concept and determine a possible crosslink between CD4+ T cells, M2 macrophages, and fibroblasts in the development of the fibrotic-inflammatory tissue in EPS.

In conclusion, a characteristic mononuclear infiltrate consisting of CD4+ cells and CD163+ cells dominates the peritoneum of EPS patients and probably drives the excessive fibrosis. These findings may suggest a role for both CD4+ T cells and pro-fibrotic M2 macrophages in the pathogenesis of EPS, although no relation with clinical outcome could be established.
